# Whey: Composition, Processing, Application, and Prospects in Functional and Nutritional Beverages—A Review

**DOI:** 10.3390/foods14183245

**Published:** 2025-09-18

**Authors:** Assiya Mirzakulova, Tolkyn Sarsembaeva, Zhulduz Suleimenova, Łukasz Kowalski, Bożena Gajdzik, Radosław Wolniak, Michał Bembenek

**Affiliations:** 1 Educational Program Group, Transport Engineering, Technology, Standardization, Certification, and Metrology, Institute of Engineering and Food Technologies, S. Seifullin Kazakh Agrotechnical Research University, Zhenis Ave. 62, Astana 010000, Kazakhstan; asiya.mirzakulova@mail.ru (A.M.); tolkyn_adil@mail.ru (T.S.); 2 Department of Food Technology and Safety, Kazakh National Agrarian Research University (KazNARU), Almaty 050000, Kazakhstan; zhulduz.suleimenova@kaznaru.edu.kz; 3 Department of Manufacturing Systems, Faculty of Mechanical Engineering and Robotics, AGH University of Krakow, A. Mickiewicza 30, 30-059 Krakow, Poland; lkowalski@agh.edu.pl; 4 Department of Industrial Informatics, Faculty of Materials Engineering, Silesian University of Technology, 44-100 Gliwice, Poland; bozena.gajdzik@polsl.pl; 5 Department of Economics and Informatics, Faculty of Organization and Management, Silesian University of Technology, 44-100 Gliwice, Poland

**Keywords:** whey, processing methods, prospects of use, functional drink, sustainable development, beverages

## Abstract

Whey is a byproduct of dairy production that possesses high nutritional value and versatile applications across various industries. In the framework of circular economy, the reutilization of whey is essential to transform this high-volume byproduct into a valuable resource. In this narrative review, the authors focus on trends in the utilization of whey for manufacturing functional and nutritional beverages. This publication summarizes the current information on whey composition based on various influencing factors and explores the technological processes currently used in whey-based beverage production and analysis, as well as nutritional profiles and properties of whey-based drinks with functional modifiers. Information about whey processing methods, compositional profiles, and whey-based beverages’ health benefits along with the author-proposed typology of functional drinks are summarized in tables throughout the paper.

## 1. Introduction

Whey is formed during the production of cheeses, cottage cheese, and other dairy products and is a byproduct with a high content of lactose, proteins, vitamins, and minerals. According to [1], the annual global production of whey is about 180–190 million tons, of which less than 50% is processed into products with practical value. The main areas of use include the production of liquid food ingredients, whey powder (about 30%), lactose and its derivatives (15%), as well as protein concentrates [2]. Modern approaches to sustainable development stimulate the processing of whey as a resource that helps reduce waste and create functional components for the food and feed industry. The scientific value of whey has been well established in numerous studies [3].

There are more than 15,000 types of whey in the world. The type of cheese made, the milk source, the amount of fat in the milk used, and the processing techniques all have a substantial impact on the composition of whey [4,5]. About 54.8 million tons of whey was generated in 2019 in the EU, leaving an excess of 13.1 million tons, which includes about 619,250 tons of lactose (4.7%). The expansion of cheese manufacturing over the past few decades has led to a notable increase in its production. Whey production is dominated by regions with established dairy industries, such as the US and EU nations. For example, the US contributes significantly to the global dairy market by processing up to 80% of its entire whey volume each year [4,6,7,8].

Only part of the whey produced in the world is reused, mainly in the form of a liquid ingredient, as well as in the form of powder, lactose, its derivatives, and protein concentrates [9,10]. The largest share of processing is accounted for by the whey produced during cheese production, which makes it the main source for obtaining food and feed components [11].

According to the latest classification, whey is a secondary dairy product obtained during the production of various types of cheeses, cottage cheese, casein, and ultrafiltrates. As a byproduct, with a high content of organic and saline substances, it is characterized by high nutritional value and has a wide range of applications. It serves as a rich source of proteins for food, biological, and functional uses, which are transferred to whey during primary milk processing. Due to its high content of organic compounds and minerals, whey is recognized as a valuable component in food technology and medical applications [12,13,14] and as a raw material for the production of food ingredients and feed additives [15,16] due to its high nutritional value, contents of lactose, easily digestible proteins, and water-soluble vitamins (B_1_, B_2_, B_6_, B_12_).

The composition of whey depends on the source product. Subcutaneous whey reflects the type of cheese and its fat content; curd whey is determined by the method of cottage cheese production and its fat content; and casein whey depends on the type of casein produced [17,18,19]. The protein fraction of whey is characterized by a favorable amino acid composition, including an increased content of branched chain amino acids. The development of processing technologies has made it possible to purposefully use whey in the production of functional foods and specialized dietary supplements. Notably, whey-derived peptides have gained significant scientific attention due to their demonstrated bioactive properties supporting muscle recovery and immune system modulation [20,21,22]. Elements like potassium, calcium, magnesium and phosphorus are included in the mineral makeup of whey. These components not only increase whey’s nutritional content but also make it a desirable component of functional foods meant to assist with cardiovascular health and bone health [23,24]. Whey from goat cheese, for example, is a premium component in specialist nutritional goods because it has higher quantities of vital amino acids and fatty acids than cow whey sourced from tropical locations. Whey composition is also affected by seasonal fluctuations, as seen by Norwegian cheddar and Dutch cheeses, whose qualities are greatly influenced by regional dairy methods [5,25,26].

Ultrafiltration, a widely adopted processing technology, effectively concentrates whey proteins and reduces non-protein components, leading to the production of high-value whey protein concentrates (WPCs) and isolates (WPIs). These products are rich in essential amino acids, making them suitable for applications in sports nutrition and dietary supplements [27,28,29].

Enzymatic hydrolysis improves the functional characteristics of whey proteins due to the controlled cleavage of peptide bonds, which leads to a decrease in molecular weight and exposure of polar groups. As a result, the solubility, emulsifying ability, and stability of the foam are improved, which makes hydrolysates promising components for functional food and beverages with immunomodulatory and regenerating properties [30,31,32,33].

Another important element that affects the uses of dried whey protein isolates and concentrates is their flavor character. By identifying the volatile chemicals that give them their sensory properties, characterization studies have assisted producers in choosing the right whey ingredients to obtain the desired product features [1,34,35].

Whey has seen creative uses in food production as a result of the increased focus on sustainability. Whey is being used more and more to make healthy and environmentally friendly products, such as fermented drinks and protein-enriched snacks. Over 90% of whey is recycled into valuable products in nations with sophisticated processing systems, including France and Germany, which set the standard for sustainable dairy industry practices [33,36].

The creation of functional beverages, like ricotta cheese whey mixed with fruit juices, is one recent advancement in the use of whey. These goods are appropriate for consumers who are health-conscious because they prolong the shelf life of whey while also improving its sensory appeal [37,38,39,40].

The question of the composition, nutritional properties, and methods of processing milk residue occupies an important place in modern scientific and technological research. For a long time, dairy waste formation was perceived as a secondary consequence of dairy processing. Recently, due to advances in technology and the expansion of biochemical research, dairy processing waste has been recognized as an important resource for the production of food, medicines, and biotechnologies [4,6,7].

The timeliness of the discussion of this issue is due to a number of circumstances. Firstly, there is an increase in demand for food products and components with a high level of nutritional properties. The whey solution includes readily available proteins, biologically active polypeptides, vitamin B components, trace elements, and sugars, which is why it is actively used in the creation of functional, athletic, and therapeutic products [11,20,24].

Secondly, the topic is closely related to the principles of sustainable development and waste-free technologies. Taking into account the huge annual production of whey, efficient processing reduces its environmental burden and at the same allows for timely obtaining economically valuable products [4,41,42].

In addition, there is an increasing interest in the scientific literature in an in-depth study of the physico-chemical and biological properties of various types of whey and their differences depending on the region, season, type of milk, and production technology. This creates a wide field for comparative analysis and systematization of information, which makes the overview format the most suitable for covering this topic [43].

Thus, the study of whey as an independent subject is an urgent area of modern science, covering aspects of food chemistry, biotechnology, ecology, and resource-saving industries.

It is necessary to also consider the aspect of circular economy in the research concerning whey and whey-based products. Currently, developments are being made in the area of biodegradable packaging options, citing biodegradability and barrier properties in the food packaging industry [44,45,46]. The packaging industry is incorporated into a larger area of research interest, namely biodegradable + recyclable polymers and polymeric composites based on whey applicable in medicine [47], the beforementioned food industry, and manufacturing (3D printing), leading to the development and popularization of sustainable and environmentally friendly manufacturing methods, such as the emulsion liquid membrane technique [48,49] or microbial fermentation [50,51,52]. Another circular economy research focus in the dairy industry is the efficient reduction of water pollution and improvement of recyclability of liquid whey waste [53]. The reutilization of waste whey can also be improved by its refinement [52,54] and modification to obtain new products [55,56,57], thus minimizing waste by increasing the byproduct’s utilization and marketability [58]. In a broader timeframe, it will be necessary to investigate the scalability of currently researched processes, as well as possibilities to integrate them in currently existing manufacturing chains.

On the basis of literature analysis, we have formulated the following research questions:

- Q1—What is the biochemical composition of different types of whey, and how do factors such as milk origin, processing technology, and regional or seasonal conditions influence its nutritional profile?
- Q2—Which technological processes are most effective for the valorization of whey into functional and nutritional beverages, and what are their respective advantages and limitations?
- Q3—How can whey-based beverages contribute to sustainable development, and what are the economic, environmental, and health-related benefits of integrating whey into circular food systems?

This review was prepared using the traditional narrative method, but it partially includes a scoping aspect as well. This decision was made to present a broad overview on the topic in question and simplify its understanding, but it also was made to include the most recent information about the article’s focus, such as applications and prospects in functional and nutritional whey-based beverages. Only peer-reviewed articles and books were taken into consideration. The databases used in research included Google Scholar, Web of Science, and Scopus, as well as private libraries of authors (printed editions). Screening of the articles was based on the relevance of the keywords and the abstract’s contents.

The scientific core contribution of this paper is its integrated approach to whey as a byproduct dairy source of colossal functional, nutritional, and environmental significance. The authors present a detailed account of the chemical composition of different forms of whey, new processing technologies (e.g., ultrafiltration, re-verse osmosis, enzymatic hydrolysis), and recent applications in food, pharmaceutical, and biotechnology industries, in the spotlight of functional beverages. The novel contribution of this review is that it combines technological, ecological, and health-focused observations and proposes directions for future research based on the principles of the circular economy. In addition, the publication provides comparative statistics on whey content by source of milk, geographical region, season, and mode of production and, thus, acts as a useful manual for systematizing scattered knowledge and establishing a basis for sustainable, high-value-added whey product production.

## 2. The Composition of Whey

The main component in the composition of whey is lactose (6.3–12.4% goes into whey), which is 70–75% in dry matter. Most carbohydrates from milk remain in whey after cheese processing, of which 90% is lactose, including small amounts of glucose, galactose, oligosaccharides, and glycoproteins [59,60,61]. The curd whey contains less lactose, due to fermentation into lactic acid, which affects the acidity of the whey. Milk fat in whey is dispersed more than in whole milk. The composition of the carbohydrate complex of whey includes monosaccharides, oligosaccharides, and aminosaccharides. Curd whey contains 0.7–1.6% glucose, which is due to the hydrolysis of lactose in the production of cottage cheese [59,62,63].

The type of milk used to make cheese has a considerable impact on the composition of whey. Thus, goat’s milk whey is characterized by an increased content of amino acids (including tryptophan and cysteine) and medium chain fatty acids (caprylic and capric), which determines its use in products for clinical and baby nutrition, as well as sports supplements [64,65]. Furthermore, the higher fat and protein content of whey made from buffalo milk affects its industrial uses [66,67].

The dry matter content and chemical composition are determined by the method and technology of primary milk processing and depend, among other things, on the type of equipment used. The water in the whey, which makes up 93–95%, exists in free and physico-chemical states according to the forms of the compound [24]. The main ones are lactose (~70%), whey proteins (~14%), minerals (~7–8%), lipids (~5–6%), and other substances (~1%), which are all values on a dry matter basis (DMB) [68].

**Figure 1:**
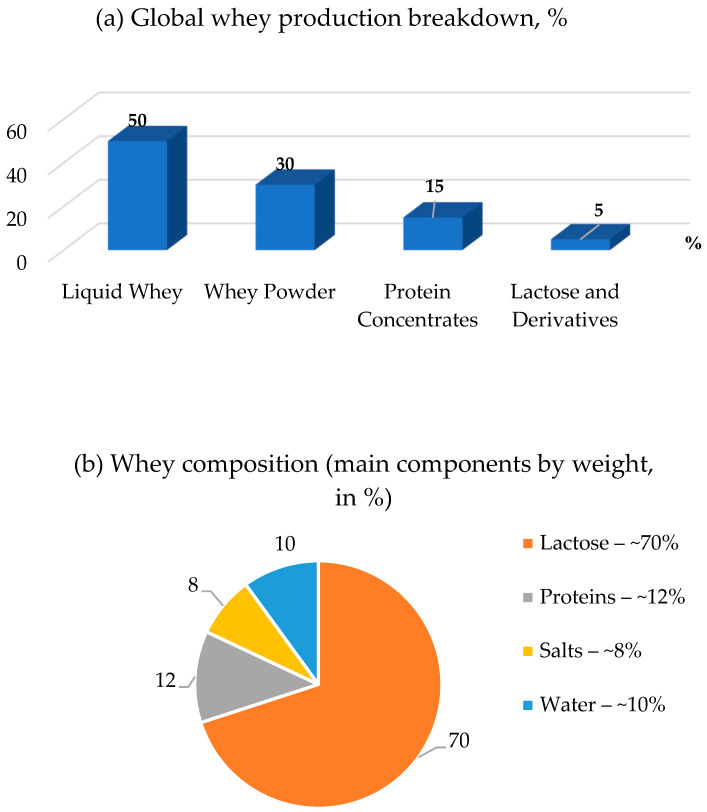

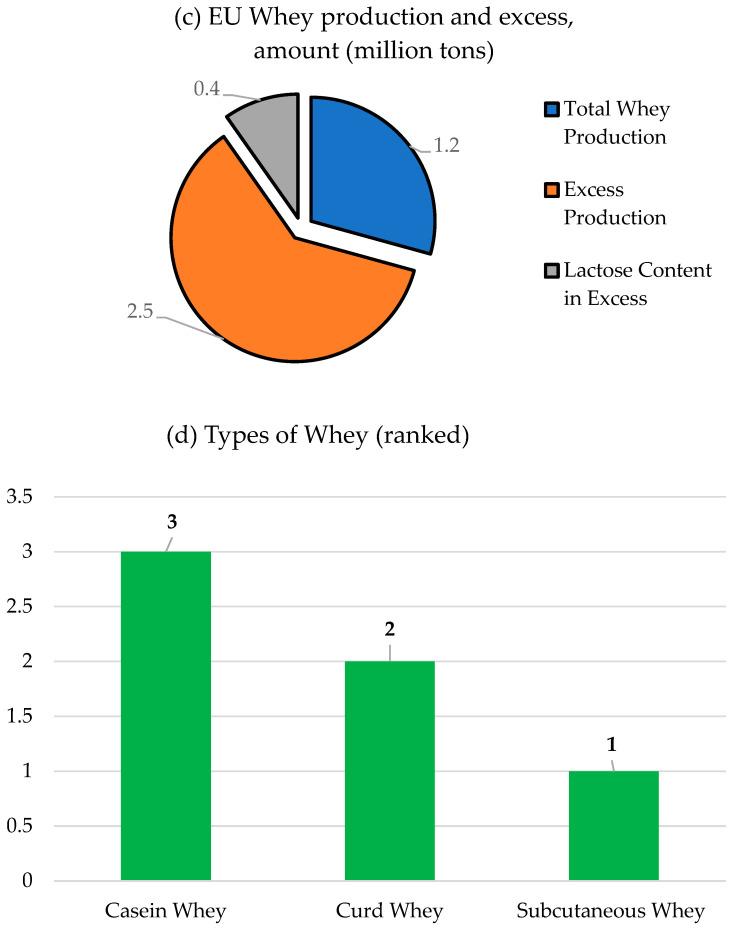
Whey production, composition, and utilization trends: (**a**) global distribution of whey production by product type; (**b**) basic composition of whey dry matter (% of major components) (note: water in liquid whey is ~93–95% and is not plotted); (**c**) EU example: total whey generated (e.g., 2019~54.8 Mt) versus unprocessed ”excess” whey (~13.1 Mt; ~24% of total); (**d**) relative prevalence of whey types by origin (sweet, acid, casein) [41,69].

Figure 1 shows various aspects of the production, composition, and effect of the milk whey. The first diagram (a) shows the distribution of whey production. The largest share, 50%, is accounted for by liquid milk whey. Second place is taken by whey powder with 30%. Protein concentrates account for 15%, while lactose and its derivatives account for only 5%. The second pie chart (b) shows the composition of whey on a dry matter basis (DMB). Lactose is the most common solid component and accounts for ~70% of whey dry matter (equivalent to ~4.5–6.0% in liquid whey, depending on type). Next are proteins, but their content is much lower.

Minerals and lipids make up smaller proportions of the dry matter; the water fraction in liquid whey (~93–95%) is not plotted [70]. Diagram (c) shows the ratio of the total volume of whey production in the European Union and the content of lactose in the unused part. Panel (c) illustrates the EU case as follows (2019): total whey generated was ~54.8 Mt, of which ~13.1 Mt (~24%) remained unprocessed (‘excess’), underscoring the need to expand processing capacity, as well as the need to optimize the recycling systems of dairy byproducts. Panel (d) summarizes the relative prevalence of whey types in production; the distribution varies by region and product mix.

From the point of view of nutritional physiology, whey is a valuable food product [71]. It contains about half of the milk’s soluble nutrients, including proteins, which make up about 20% of milk proteins, milk sugar, mineral salts, and water-soluble vitamins [72].

The most important vitamins that pass from milk to whey are riboflavin, folic acid, and cobalamin. The latter two are associated with whey proteins and pass into whey during cheese production. MS contains more vitamin B_2_ than milk due to the activity of lactic acid strains used in cheese production. Due to the relatively high level of riboflavin, the whey has a characteristic yellowish-green color [42,73]. Table 1 presents the compositional characteristics of different whey types.

The comprehensive analysis of whey components, as presented in Table 1, reveals significant variations in protein, carbohydrate, mineral, and vitamin profiles across different whey types, reflecting their distinct cheese-making origins and functional applications in food and nutraceutical industries [74].

**Table 1:** Comprehensive compositional profile of whey types.

Component	Sweet Whey (Hard Cheese)	Acid Whey (Soft Cheese)	Mediterranean Whey	Notes and Biological Significance	Source
PROTEINS and AMINO ACIDS					
Total protein (%)	0.8–1.0	0.5–0.7	0.6–0.9	Higher BCAAs ^1^ in sweet whey	[42,73]
Free amino acids	4 × milk levels	10 × milk levels	6–8 × milk levels	Bioactive peptide precursor	[12]
CARBOHYDRATES					
Lactose (%)	4.5–5.0	5.5–6.0	4.0–4.8	Higher in acid whey	[3,62]
Oligosaccharides	Present	Elevated	Variable	Prebiotic effects	[75]
FATS					
Fat content (%)	0.05–0.3	0.1–0.5	0.2–0.4	Impacts creaminess in applications	[76]
MINERALS					
Anions (PO_4_^3−^, Cl^−^, citrate, g/L)	5.831	6.120	5.450	Impacts electrolyte balance	[33]
Cations (K^+^, Na^+^, Ca^2+^, g/L)	3.323	3.850	3.500	Ca^2+^ higher in acid whey	[18]
Trace elements (mg/100 g)					
-Zinc	0.3108	0.2800	0.2950	Immune function support	[18]
-Iron	0.0674	0.0721	0.0690	Bound to lactoferrin	[18]
VITAMINS (% transfer from milk)					
B vitamins (B_1_/B_2_/B_12_)	81/91/58	75/85/50	78/88/55	Heat-stable in sweet whey	[77]
Fat-soluble (A/E)	11/32	8/28	10/30	Lower transfer due to lipid removal	[78]

^1^ BCAA—branched chain amino acids.

The data reveal that sweet whey contains higher protein levels (0.8–1.0%) and superior retention of B vitamins (e.g., 91% riboflavin), making it ideal for nutritional supplements. In contrast, acid whey is richer in lactose (5.5–6.0%) and minerals like calcium, while Mediterranean whey shows a balanced profile suitable for traditional dairy applications. These compositional differences directly influence their functional uses in food technology and health products [79]. Figure 1 shows the percentage composition of whey components.

Regional and seasonal variables have a big influence on the composition of whey. Whey often has higher fat and protein contents in colder climates because the nutritional composition of milk changes over the winter. The composition of whey is also significantly influenced by regional practices, such as providing particular diets to animals. For instance, Norwegian Dutch-type cheese whey typically exhibits higher concentrations of calcium (~120 mg/100 g) and phosphorus (~95 mg/100 g) than cheddar cheese whey, due to the mineral retention during cheddar’s acidic precipitation process [28]. In contrast, southern European whey (in comparison to northern European variants, e.g., from Italian mozzarella or Spanish Manchego) shows elevated levels of potassium (~180 mg/100 g) and magnesium (~12 mg/100 g), reflecting the use of buffalo/goat milk in some traditions. Warmer climate diets affect milk composition and alkaline brine-washing techniques that selectively preserve these minerals [80,81].

## 3. The Use and Benefits of Milk Whey

**Figure 2:**
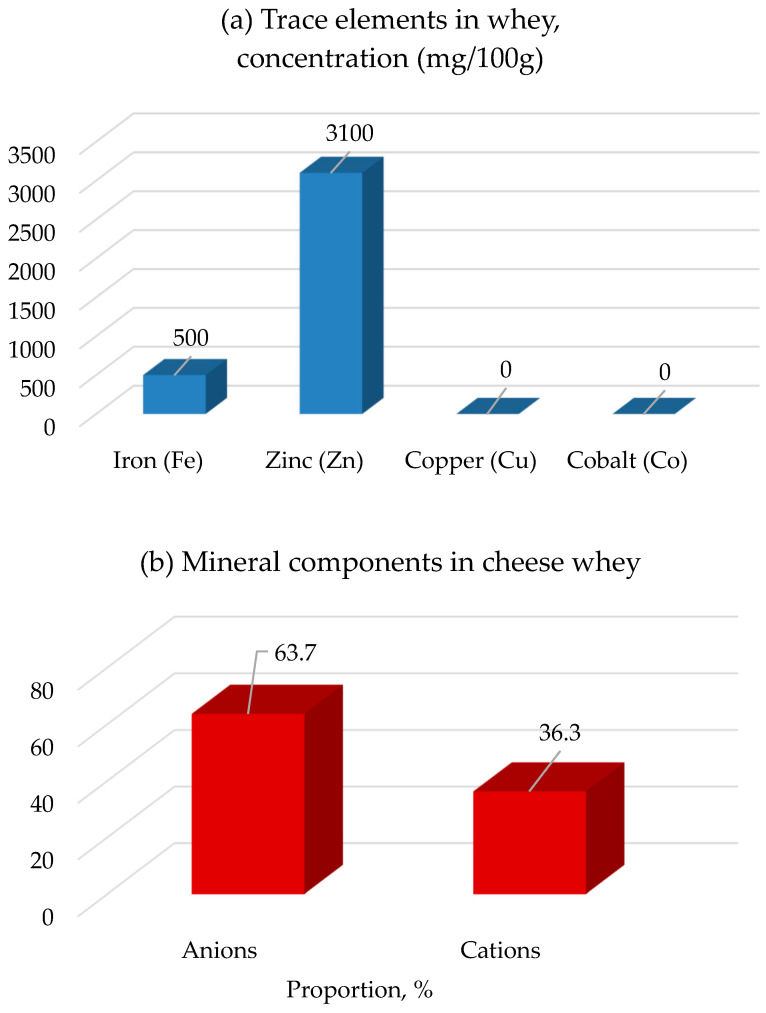
Mineral and trace element composition of whey types: (**a**) concentration of trace elements (Fe, Zn, Cu, Co) in acid whey, expressed in mg/100 g (values approximate, vary by source); (**b**) distribution of mineral components (cations and anions) in sweet whey, expressed as % of total mineral fraction. Data compiled from multiple sources [18,33,61,79].

The nutritional value of whey is characterized by the following full set of indicators for food products: high quality, high calorie content, good digestibility, sufficient nutrient content, and biological and physiological usefulness. All over the world, there is a positive trend in the volume of whey intended for processing due to an increase in the production of cheese and casein [42,75]. Figure 2 illustrates the trace element and mineral composition of whey.

Figure 2a illustrates the approximate concentration ranges of trace elements (Fe, Zn, Cu, Co) in acid whey. Zinc typically shows the highest concentration (up to ~3 mg/100 g), while iron is present at lower levels (~0.5 mg/100 g). Copper and cobalt are usually either not detected or occur in trace amounts below analytical sensitivity. These values vary depending on milk origin, processing technology, and seasonal factors; therefore, the figure presents ranges rather than exact values. Figure 2b presents the distribution of mineral components in sweet whey, grouped into cations and anions. Anions account for ~60–65% of the total mineral fraction, and cations for ~35–40%. The precise ratio depends on the cheese-making process and regional milk characteristics. Key cations include K^+^, Na^+^, and Ca^2+^, while major anions are phosphate, chloride, and citrate. The composition, as well as the high production volumes associated with the impact of whey on the environment, leads to great interest in the increase of its full utilization. Thus, the study of this byproduct for possible future use is of significant importance [82].

Whey consists of 93–95% water; the remaining part includes carbohydrates, proteins, vitamins, and minerals. Despite its low energy value (about 24 kcal/100 g), it has a high biological value due to the content of easily digestible proteins, lactose, vitamins (A, groups B, C, E), and minerals (Ca, K, Mg, Fe). These characteristics determine its use in the production of dietary and specialized food products [83,84].

Given the high biological value of whey, as well as significant volumes of its production, it is difficult to achieve high profitability of production without its rational use. The high nutritional and biological value of whey is well known and is due to the presence of valuable carbohydrates, minerals, enzymes, phospholipids, vitamins, organic acids, and easily digestible whey proteins, such as albumins and globulins. Whey is used in the production of children’s and therapeutic nutrition products with reduced energy value, including beverages with fruit and vegetable components, as well as protein products with high nutritional value. As a result of studying the properties and significance of whey, along with the needs of baby food products, it can be argued that whey not only has a composition close to mother’s milk, but it also significantly affects the production process of baby food. In particular, the addition of whey to infant formula simplifies production by providing a balanced source of essential protein, lactose, and minerals that accurately mimic the nutritional profile of breast milk. This reduces the need for additional enrichment and improves product quality while reducing production costs. Its economic profitability is due to its wide availability as a byproduct in the dairy industry and its ability to replace more expensive ingredients without compromising nutritional value [76,85,86].

Gelation, emulsification, and foaming are among the functional characteristics of whey proteins that are well known. Because of these qualities, whey is a crucial component in the creation of functional meals like protein bars, sports drinks, and desserts made with dairy. There is much promise for improving cardiovascular health and controlling blood pressure with the bioactive peptides produced from the hydrolysis of whey protein [43,87].

Whey is widely used in various industries due to its nutritional and functional properties. In the food industry, it is used in the production of bakery and confectionery products, where it improves texture, moisture retention, and shelf life. In ice cream, whey is a source of lactose and protein, increasing creaminess and reducing crystallization. Processed cheese benefits from whey because it acts as an emulsifier and improves melting. In the pharmaceutical and cosmetic industries, milk whey is added to medicines and skin care products because of its biologically active compounds that help moisturize and restore the skin. In addition, whey is used in the production of animal feed, providing an economical and protein-rich supplement that supports the growth and health of animals. Research highlights its role as a versatile material in these applications, making it a key component in various technological processes [88,89].

Whey proteins and oligosaccharides are used in infant formulas to approximate their nutritional profile to breast milk, as well as to support the formation of healthy intestinal microflora in newborns [79,90].

It is widely used for the manufacture of vaccines in pharmaceuticals [91], as well as in medicine. Most widely, this product is used in the native field of production, for the manufacture of dairy products. However, for all the advantages of whey and products derived from it, in the field of processing secondary dairy raw materials, the task aimed at its full use in food products has not yet been fully solved. Therefore, dairy production technologists have the opportunity to create dairy products based on whey. Whey-based drinks are considered as a promising form of specialized nutrition, including for the elderly and patients with water restriction. Due to regulated moisture content and low osmolarity, such products help maintain hydration and ensure the intake of easily digestible nutrients [92,93].

As a person ages, resulting in decreased receptor susceptibility, loss of sensitivity to thirst develops, and fluid deficiency can lead to dizziness, confusion, heart and vascular diseases, loss of muscle mass, and reduction of protein and glycogen reserves [82,94].

Whey has many applications as an independent drink (fresh or with the addition of juice). In medicine, milk whey is used in the treatment of various diseases due to its beneficial properties. For example, it has the same effect as insulin in the treatment of diabetes, enriches the body with necessary macro- and microelements, helps the digestive system recover, and supports recovery in pancreatitis. In addition, whey improves the functioning of the colon, relieves inflammatory processes, and improves liver function, which makes it an important component of therapeutic nutrition. Whey has a beneficial effect on blood circulation, helps normalize blood pressure, lowers cholesterol, and cleanses the body of toxins [84,85].

It is recommended for use with people suffering from cardiosclerosis, atherosclerosis, gastritis, and pancreatitis. It proves to be an effective supplement food product for older people, helping to speed up metabolism and make up for the lack of nutrients that cannot be obtained from other sources at this age stage.

Beyond its use in food, whey also contributes to sustainability in non-food industries. Whey, for example, is being utilized more and more to produce biofuels and bioplastics, which lessens the need for non-renewable resources. Whey fermentation has also been found to be a feasible renewable energy source for producing hydrogen, with major environmental advantages [31,72,95].

## 4. Technology of Production and Processing of Whey

A useful byproduct produced during the manufacturing of several dairy products, such as cheese, yogurt, and casein, is whey. The type of milk used and the production technique determine the composition and characteristics of whey. For instance, the compositions of acid whey, a byproduct of the creation of lactic acid-coagulated cheese, and sweet whey, which is formed during the rennet-coagulated cheese-making process, are very different. Similarly, whey from ultrafiltered milk used in yogurt production contains higher protein concentrations [96,97,98].

The efficient use of whey has been made possible by modern processing technology. Proteins can be concentrated and unwanted substances like lactose and minerals can be eliminated using membrane filtering techniques like ultrafiltration, nanofiltration, and reverse osmosis. Ultrafiltration is one of the key methods used to obtain whey protein isolates and concentrates, which are used in sports nutrition and functional foods [99]. Various methods used for processing whey and manufacturing whey products are presented and summarized in Table 2. The methods presented can be applicable at each stage of the manufacturing process, improving the composition and properties of the product.

The most effective methods of whey processing include concentration, thickening, and drying. These technologies make it possible to obtain products from whey with high nutritional and biological value, extended shelf life, and optimal technological properties. Concentration and ultrafiltration separate proteins and lactose to produce high-protein products such as isolates and whey protein concentrates, which are used in functional foods and beverages. Drying, often carried out by spray drying, turns liquid whey into powder, which facilitates storage and transportation [102,103,104].

With the development of new technologies and awareness of the beneficial properties of whey, its use in the food industry will expand, making it part of modern food production. Currently, in industrialized countries, 70–90% of whey is processed for food purposes. However, the problem of the complete and rational use of whey has not yet been solved worldwide. Therefore, research on the development of new types of products using whey is relevant [101,105].

The problem of the reasonable use of whey exists in all countries with a high stage of development of the dairy industry. Domestic production processes about 20%. In many industries, the remaining whey, which makes up the majority, is discharged into the sewer system without cleaning, causing serious damage to the environment. This practice is mainly due to the lack of advanced processing technologies, the lack of economic incentives, and the high cost of building processing plants. Unprocessed whey contains a significant amount of organic substances, mainly lactose and proteins, which, when released into water bodies, contribute to the biochemical consumption of oxygen and can cause an ecological imbalance. Studies emphasize the urgent need for sustainable solutions to address this issue and highlight successful examples in countries like France and Germany, where up to 90% of whey is processed into valuable products. More and more attention is being paid to the processing of whey. In countries with a modern technological base, such as France, the USA, Sweden, Germany, etc., in order to maximize the value of whey, 70–90% of it is processed using advanced technologies. The difference in treatment rates depends on factors such as the size of the dairy industry, the availability of treatment facilities, and economic incentives for waste management. The introduction of whey processing technology can not only reduce emissions of pollutants into the environment, but it can also contribute to the sustainable and waste-free production of other resources. This approach increases the efficiency and profitability of the dairy industry by converting food products into agricultural protein, food products, functional beverages, etc. [106].

The processing technique determines the kind of whey byproduct that is produced. For instance:

- Cheese whey is perfect for creating highvalue protein concentrates, as cheese whey has higher protein and fat concentrations than other forms of whey [82,92].
- Acid whey is made from the manufacturing of cottage cheese and yogurt. Compared to cheese whey, acid whey has a lower protein content but is higher in lactose and minerals, which limits its use in industry [59].
- Casein whey is suitable for certain uses, such as lactose synthesis, as it usually contains less fat and lower levels of calcium and phosphate but higher chloride content than cheese whey [88].

Worldwide interest in whey continues to grow. First of all, this concerns the technologies of the deep processing of whey (e.g., enzymatic hydrolysis, membrane filtration, chromatographic separation), which make it possible to obtain products similar to medicines. Many methods of the deep processing of whey have not found their application in practice due to economic indicators and low profitability. Taking into account the above facts, it can be concluded that, at the moment, the search for and development of a suitable deep processing of whey, as well as the development and production of dairy products using whey, are relevant. The problem of whey processing can be solved by producing a variety of drinks based on it. These drinks are popular with both producers and consumers. To increase the biological and nutritional value, vitamins, proteins, and herbal extracts of medicinal plants with high antioxidant properties can be added to drinks [94,107,108].

The incorporation of sustainable techniques, like closed-loop systems, which recycle more than 90% of whey wastes into useful products, is one example of recent developments in processing technologies. These systems optimize resource efficiency while minimizing their negative effects on the environment. The capacity to separate particular components, such as glycomacropeptides and immunoglobulins, has been further improved by emerging techniques, including microfiltration and chromatographic separation [109,110,111].

Innovations in whey processing have been fueled by the rising demand for sustainable food production. For instance, in their individual marketplaces, whey-derived goods like bioplastics and protein-enriched drinks are becoming more and more well-liked as environmentally friendly substitutes. To increase the uses of whey in the food and non-food industries, researchers are still investigating biotechnological approaches and enzymatic modifications [77,112].

## 5. Application of Whey in Functional Beverages

Whey, as a byproduct of milk processing, contains many components in its composition. After separation of the protein fraction, 15–25% of proteins, up to 95% of lactose, almost all trace elements, and most of the vitamins of milk remain in the milk whey. In many countries with a developed dairy industry, the full and rational use of whey is carried out. The milk whey is drained as waste or, at best, used to fatten farm animals. Solving the problem of the full and rational use of whey will allow us to obtain a pronounced economic, environmental, and medico-social effect for the region. The processing of secondary dairy raw materials at enterprises of the cheese industry using the presented technology acts as an additional source of profit [113,114].

The development of beverages made with whey lessens the environmental effect of whey disposal, which promotes sustainability. Whey is converted into drinks with additional value rather than being disposed of as trash, which is consistent with the ideas of the circular economy. Businesses in North America and Europe have embraced cutting-edge processing technology to reduce waste and optimize resource use [8].

This transformation of whey from a disposal problem into a resource for value-added products exemplifies the principles of a circular economy, where waste streams are reintegrated into production cycles. Furthermore, whey utilization helps reduce greenhouse gas emissions from dairy effluents and contributes to sustainable food system strategies adopted in the EU and North America. Several environmental life cycle assessments (LCAs) have shown that valorizing whey significantly improves the ecological footprint of dairy operations [31,83,115].

The production of whey drinks is a key focus for many countries with a developed dairy industry. In these regions, whey is processed into valuable products such as functional drinks, protein powders, and lactose derivatives. The policy of many countries in the field of healthy nutrition gives priority to the development of technologies for the production of functional foods, including products based on whey. These efforts are aimed at maximizing the economic, environmental, and health–social benefits of whey processing. Functional whey drinks enriched with amino acids, vitamins, and plant-based components offer biologically complete nutritional solutions and satisfy the growing consumer demand for health-oriented products [116,117].

In recent years, interest in functional foods that contribute to the preservation of health and the prevention of diseases caused by inadequate and unbalanced nutrition has increased worldwide. Biotechnological approaches to the production of safe food products are actively used in Europe; currently, 25% of the diet in Europe and 60% of the diets in the USA and Japan are fermented foods [67,96,118]. In recent years, significant attention around the world has been focused on the development of energy drinks using various qualities from various substrates of both dairy and non-dairy origin, and this has been proven by numerous studies and innovations in this field. Currently, the production of functional products, including beverages, is becoming relevant, which provides for the use of whey, which contains a complex of biologically active substances, as one of the main components [69,119,120].

Due to customer desire for sustainable and health-conscious products, the market for whey-based functional drinks has grown significantly in recent years. Younger customers favor high-protein beverages with natural ingredients and clean labels, according to surveys. Manufacturers have responded by concentrating on creating novel whey drinks with clean packaging and less processing [106,121].

Whey-based functional beverages are becoming more and more well-liked because of their many health advantages and high nutritional content. These drinks are full of minerals, bioactive peptides, and important amino acids that support the immune system, gastrointestinal health, and muscle recovery. To improve their functionality and attract health-conscious consumers, whey-based drinks are frequently fortified with extra nutrients, including vitamins and probiotics [122].

Protein shakes, smoothies, and fermented drinks are just a few of the many whey-based beverages that have been made possible by recent developments in product formulation. Whey-fermented drinks enhanced with kefir grains, for instance, appeal to a wide variety of customers due to their distinct sensory qualities and probiotic advantages [123]. Additionally, drinks with better nutritional profiles and tastes have been produced by combining whey with fruit juices [77].

To provide a clearer structure, the various types of whey beverages, including those enriched with fillers, superfoods, and probiotics, are discussed in detail in the following subsection.

### Whey Beverages with Fillers and Additives

This subsection focuses on whey drinks enriched with various natural fillers and functional additives, which improve nutritional content, sensory qualities, and stability.

In order to improve whey drinks’ nutritional content, sensory qualities, and shelf life, fillers and additives are essential. Natural fibers like pectin and inulin, which enhance texture and offer prebiotic advantages, are frequently used fillers. While keeping a clean-label profile, additives like fruit purees, natural flavors, and sweeteners like honey or stevia improve taste [69,97,110]. Additionally, whey drinks are purposefully enriched with herbal components of local juicy vegetable raw materials, which will expand the range of products.

To satisfy particular nutritional requirements, whey drinks are frequently enhanced with vitamins, minerals, and bioactive substances. For example, omega-3 fatty acids and antioxidants like vitamin C or E are included for cardiovascular benefits, while calcium and magnesium are added to promote human health. To improve gut health, probiotic strains like *Lactobacillus* and *Bifidobacterium* are also commonly introduced [124,125]. The high biological value of whey is determined by its content of protein, carbohydrate, and lipid complexes. Whey’s diverse composition includes biologically active substances such as minerals, vitamins, organic acids, amino acids, carbohydrates, and enzymes, which are integral to its functional properties.

The incorporation of superfood ingredients like chia seeds, matcha, and turmeric in whey drink formulas is one recent innovation that enhances both nutritional value and consumer appeal. To achieve a creamy texture without the use of artificial stabilizers, natural thickeners such as guar gum and xanthan gum are employed. The increasing demand from consumers for natural and functional beverages is in line with these developments [111,118,124,126,127].

Studies highlight that the use of secondary dairy raw materials, produced in significant quantities, increases the nutritional value of beverages and reduces production costs. The high biological value of whey is determined by its content of protein, carbohydrate, and lipid complexes. Whey’s diverse composition includes biologically active substances such as minerals, vitamins, organic acids, amino acids, carbohydrates, and enzymes, which are integral to its functional properties [60,89]. For example, advanced whey processing technologies, such as ultrafiltration and reverse osmosis, enable the retention of essential nutrients while improving economic efficiency [31,114,128].

Because functional and fortified beverages are becoming more and more popular, the market for whey drinks with fillers and additives is still growing. Drinks that mix delicious flavor and health advantages are becoming more and more popular, which is pushing producers to try out novel flavor combinations and useful components. Furthermore, the preference for natural additives over synthetic ones has been impacted by legal requirements for clean-label products [17,19].

On the basis of analysis, the authors have proposed the typology of functional whey-based beverages (Table 3). This functional whey drink typology is a novel taxonomy blending nutritional functionality, food processing technology, and consumer-protected needs. In contrast to the conventional compositional snapshot, this approach envisions a matrix scientifically correlating major functional components to respective target groups and technology approaches. This typology set out systematically not only makes summarizing current knowledge status possible but also proposes priority directions for innovation and product development in the dairy and functional beverage sectors.

The typology follows the dominant bioactive constituents that define the beverage’s beneficial health impact. Thus, probiotic-enriched beverages are designed to stabilize the intestinal microbiota and enhance gut health, a need consistent with global trends in preventive nutrition. Similarly, amino acid-enriched drinks with high concentrations of branched-chain amino acids (BCAAs) are designed for muscle recovery and protein supplements in sports individuals and active consumers. This highly specialized functional profile enables scientists and formulators to optimize levels and composition of nutrients to precise physiological needs.

The consumer grouping segmentation identifies that functional drinks are heterogeneous in character and not homogeneous and rather have to satisfy the segmented needs of various segments of society. Infants and children, for example, are fed goat whey-based foodstuffs due to their hypoallergenic character and rich content of cysteine. The elderly require high bioavailability beverages of calcium and B vitamins for bone mineral content and metabolic functions. The market penetration of student and cognitively active consumer products (e.g., energy-fortified whey drinks) indicates expansion prospects in developing economies in functional drink markets outside of mainstream sports or clinical segments.

The typology also relates each beverage category to the appropriate processing technology, and method–function synergism is required. Ultrafiltration or enzymatic hydrolysis, for example, are not technology choices; these determine the physicochemical profile and bioavailability of main constituents. Enzymatic hydrolysis, for example, improves digestibility and liberates bioactive peptides with antioxidant or immunomodulating activity, whereas spray drying gives long shelf-life and distribution convenience. This indicates the requirement for accuracy in choosing technology against both dietary requirements and expense.

The sensory profile section introduces the straightforward, but often overlooked, element of consumer acceptability. Functionality for health is necessary, but palatability and sensory acceptance are major drivers of human acceptability and business viability. Lightly carbonated fermented whey drinks can be a hit among the probiotic-conscious consumer, but human-designed, mild, sweet, and smooth textures can be best positioned in pediatrics. The “clean label” and natural flavor movement of consumers also holds in favor of the utility of botanical and herbal infusions as functional and sensory modifier agents.

## 6. Modern Trends in Enhancing Whey-Based Beverages

One of the important directions in the field of healthy nutrition is the development of technologies for the production of functional products that promote health. As part of scientific research in the food industry, new technologies are being actively created, including products in which recycled raw materials are combined with various additives, including those of plant origin. Recently, products have been created in which the secondary base is combined with various additives, including those of plant origin. This ensures a high level of nutritional balance in terms of amino acid and vitamin compositions, as vegetable raw materials are rich in trace elements and vitamins, complementing the nutritional profile of whey [40,127].

The use of non-traditional raw materials—such as plant-based extracts (e.g., chamomile, mint, rosehip), insect proteins, algae, and fermented cereals—for dairy products is one of the promising directions for increasing the nutritional and biological value of combined products. These ingredients enhance functional properties (e.g., antioxidant activity, prebiotic effects) while aligning with clean-label trends [4,87].

The use of whey-based products as vegetable fortifiers to produce a combined product has broad prospects in the production of whey-based products [65]. Currently, much attention is being paid to the development of products for therapeutic and preventive nutrition, enriched with various biologically active substances, including vitamins. Such products are obtained by aiding in the introduction of enriching additives of plant origin into mass-consumption food products. Among the various types of dairy raw materials, a special place is occupied by whey, which can serve as a good basis for creating new generation products; the composition of whey allows you to create a product with high nutritional and biological value. The summarization of selected additives and desired properties of whey-based products is presented in Table 4.

The use of these additives makes it possible to create functional whey products that combine high nutritional value with targeted health benefits. For example, whey-based beverages enriched with extracts of medicinal plants such as chamomile, mint, or rosehip help strengthen the immune system and possess antioxidant properties. These products are popular for their ability to address vitamin deficiencies and improve overall health [105,139,140].

Whey-based products are in demand due to the use of environmentally friendly raw materials and a relatively low cost compared to traditional animal proteins, which increases their economic attractiveness [131,139,141].

The problem of the prudent use of whey exists in all countries with a developed dairy industry, regardless of the form of ownership and the system of economic relations. The rational use of whey largely determines the efficiency of both individual enterprises and the entire dairy industry as a whole. In order to ensure the resource conservation and environmental safety of dairy production, both at the moment and in the future, it is necessary to target enterprises to fully involve whey resources in industrial processing [3,142,143].

## 7. Discussion on Methods and Standards in Compositional Analysis of Whey

To ensure the high quality of whey, it is important to use accurate and reliable analysis methods that allow for monitoring the basic physico-chemical parameters. Some of the key parameters to be monitored are the mass fraction of dry substances, the sodium chloride content, and the lactose level [60,141,142].

Various types of refractometers are widely used to determine the mass fraction of solids in whey. These devices are based on measuring the refractive index of a liquid, which correlates with the concentration of dissolved substances. The refractometric method is characterized by high measurement speed, ease of operation, and sufficient accuracy, which makes it a convenient tool for the regular monitoring of product quality [9,144].

The sodium chloride content in the serum (defined as waste byproduct liquid of whey protein concentration process) is determined by the conductometry method based on measuring the electrical conductivity of the solution. This method provides rapid control of the salt composition in both laboratory and industrial conditions, including a wide range of concentrations [42,145].

The mass fraction of lactose is determined using saccharimetry and spectrophotometry. The first method is based on measuring optical activity, while the second is based on recording light absorption at a certain wavelength. Both approaches ensure high accuracy of analysis and are used to assess the quality of raw materials in the production of powdered dairy products, baby food, and functional additives [116,146,147,148].

The introduction of modern standards for the quality control of whey contributes to improving the reliability and accuracy of analytical studies. An integrated approach to analysis includes not only methods for determining key components but also the regulation of the processes of acceptance, sampling, and processing of results. Due to this, manufacturers can ensure stable product quality and compliance with regulatory requirements [149,150].

The use of modern quality control methods helps to ensure stable technological parameters during the processing of whey and reduces the likelihood of deviations in the composition of products. This increases production efficiency and makes it possible to meet established quality and safety standards, which is especially important in the context of the intensification of the dairy industry [149,150].

Internationally, whey quality management is greatly aided by ISO standards. For example, ISO 8968-1:2014 [151] focuses on determining the nitrogen content to evaluate protein quality, while ISO 22662:2024 [149] describes techniques for quantifying lactose content using high-performance chromatography. Even with these set criteria, there are still issues. These compositional differences directly influence their functional uses in food technology and health products [65], such as the lack of uniform measurement methods for certain quality parameters, highlighting the need for further research and standard development. Incorporating these standards into the whey industry promotes better product quality and safety while providing clear benchmarks for trade [149,151,152].

The European Union alone generates substantial excess whey, underscoring the need for advanced processing technologies and innovative uses to minimize waste and maximize its potential. Whey’s versatility—from its nutritional role in protein supplements to its functional applications in medical fields—demonstrates its importance in fostering sustainability and optimizing resource utilization. As demand for high-quality proteins and functional foods continues to rise, whey stands as an essential contributor to meeting these challenges [10,108,136].

Traceability and sustainability are increasingly being incorporated into whey production methods. To ensure accountability and transparency, many producers have implemented traceability systems that follow whey from raw milk to the final product. Whey processing facilities are increasingly adopting sustainable methods, like using renewable energy and cutting waste, in line with international initiatives to lessen their negative effects on the environment [82,93,98].

## 8. Conclusions

The biochemistry of the varied sources of whey—i.e., acid whey, sweet whey, and casein whey—is still somewhat variable with respect to the milk source, cheesemaking, and climatic factors. Sweet whey, which is generally a byproduct of hard cheeses due to the application of rennet, has more protein (0.8–1.0%), branched-chain amino acids, and up to 91% of the B vitamins of milk like riboflavin. Conversely, whey acid from the process of preparing soft cheese or yogurt has a higher percentage of lactose (5.5–6.0%) and minerals, i.e., phosphate and calcium. Seasonal and geographical considerations have an effect on this profile, too; goat or buffalo milk whey, for example, under suboptimal warm weather conditions, will contain more medium-chain fatty acids and essential amino acids, whereas cold climate counterparts—e.g., Norwegian cheddar-type whey—will have more calcium and phosphorus due to specific animal feeding and coagulation procedures. Process technologies like ultrafiltration or spray drying control nutrient retention by concentrating protein and removing lactose or minerals, thus dictating the end nutritional and functional characteristics of whey-containing foods (Q1).

The most favorable technological technologies for whey valorization into nutritional and functional beverages are ultrafiltration, reverse osmosis, nanofiltration, enzymatic hydrolysis, and spray drying. Ultrafiltration has seen extensive use in whey protein concentration and the removal of low-molecular-weight components for the production of high-protein isolates and concentrates for applications in diet and sport foods, but it requires high-technology membrane equipment and is susceptible to fouling. Reverse osmosis easily scale-concentrates the water content on to cheaper transport and storage but is typically limited to pre-filtered whey streams. Salts and lactose are selectively rejected by nanofiltration to allow the manufacture of lactose-free drinks at the expense of expensive apparatus. The hydrolysis of enzyme protein structure increases digestibility, solubility, and biofunctionality—particularly immunomodulatory and antioxidant functions—but has to be carried out under the extreme control of enzymic conditions and processed more elaborately. Spray drying to obtain powder from liquid whey improves shelf life and logistics ease but is energy-expensive. These technologies by themselves, or combined in synergy, allow the creation of value-added beverages through the virtue of health trends, clean-label demands, and sustainability objectives (Q2).

Whey drinks help to achieve sustainable development by upgrading a long-time undervalued byproduct into a value-added functional food and therefore achieving the principles of the circular economy. From an economic perspective, the use of whey helps to save on waste treatment and create new sources of revenues for the dairy sector through the substitution of more costly raw materials with nutritionally equivalent alternatives. Ecologically, whey valorization avoids the environmental load of nutrient-rich and organic raw milk effluent staying around by switching it to harmless, edible molecules and reducing greenhouse gas emissions and eutrophication potential. From a health perspective, whey beverages provide essential amino acids, vitamins, minerals, and bioactive peptides to support immune system function, muscle integrity, and metabolic control. Their fortification with herb extract, superfood, and probiotics also makes them attractive to health-aware consumers. Therefore, the overall application of whey in manufacturing drinks not only leads to conservation and waste reduction but also to the economic sustainability of agricultural food systems as well as public well-being (Q3).

Dairy whey, once regarded as waste, is now a versatile raw material with applications spanning food, medicine, cosmetology, and agriculture. Advanced technologies enable the extraction of high-quality proteins, lactose, minerals, and bioactive compounds, aligning with demands for sustainable and health-focused products [8,22,33,115,134].

Historically, the majority of whey was discarded or used as low-value animal feed, resulting in environmental burden and economic inefficiency [4,6]. Its high organic load made it a pollutant when untreated. However, early studies began to highlight its protein quality and potential in food applications [41,69].

Currently, modern processing techniques such as membrane filtration, enzymatic hydrolysis, and chromatographic separation have made it possible to efficiently extract valuable components from whey [30,77,109]. Functional whey-based beverages, including those enriched with probiotics, superfoods, and herbal additives, are being developed to meet the growing demand for clean-label, high-protein, and health-promoting drinks [94,96,103]. The beverage sector, in particular, has seen rapid innovation due to whey’s compatibility with both nutritional and sensory requirements.

Looking forward, the future of whey utilization lies in biotechnology-driven solutions such as precision fermentation, smart packaging, AI-based process optimization, and incorporation into circular bio economy models. Emerging applications include biofuels, biodegradable plastics, and highly targeted nutraceuticals [31,83,115,143].

The rational utilization of whey reduces environmental burdens while enhancing the dairy industry’s economic viability. Innovations in processing have paved the way for sustainable and functional whey-based products, meeting both industry and consumer needs. By continuing to develop efficient, eco-friendly, and value-added uses for whey, the dairy sector can evolve toward a more circular and resource-resilient future [19,99,144,153].

## Figures and Tables

**Table 2:** Comparison of whey processing methods.

Method	Purpose	Advantages	Challenges	Applications	Sources
Spray Drying	Convert liquid whey to powder	Long shelf life, easy transportation	High energy consumption (~5000 kJ/kg)	Powdered milk, protein supplements	[93,100]
Ultrafiltration	Separate proteins and lactose	Produces high-protein concentrates	Requires advanced equipment (membrane fouling risks)	Functional foods, sports nutrition drinks	[30,94,101]
Nanofiltration	Extract salts and lactose	Enhances product purity	High initial setup costs	Lactose-free products, infant formulas	[38,94,101]
Reverse Osmosis	Reduce water content	Energy-efficient, reduces transportation costs	Limited to pre-filtered whey streams	Pre-concentration for drying	[31]

**Table 3:** Typology of functional whey-based beverages.

Type of Whey-Based Beverage	Dominant Functional Component	Target Consumer Group	Processing Technology	Sensory Profile
Probiotic (e.g., whey kefir)	Probiotics (Lactobacillus, Bifidobacterium)	Individuals supporting gut microbiota	Fermentation with starter cultures	Sour, refreshing, lightly carbonated
Amino acid-enriched (for athletes)	BCAAs, leucine, glutamine	Athletes and physically active individuals	Ultrafiltration + amino acid fortification	Neutral, milky, slightly sweet
Mineral–vitamin drink (for seniors)	Calcium, magnesium, vitamins B, D, E	Seniors and individuals with deficiencies	Mineral enrichment + spray drying	Delicate, mildly sweet, herbal or mineral-like
Herbal whey beverage	Polyphenols, flavonoids, essential oils	Consumers preferring natural, plant-based ingredients	Herbal infusion + pasteurization	Botanical, slightly astringent, aromatic
Goat whey drink (for children)	Medium-chain fatty acids, cysteine	Infants, children, people with casein intolerance	Enzymatic hydrolysis + composition standardization	Mild, slightly sweet, smooth
Energy drink (with caffeine and BCAAs)	Caffeine, taurine, amino acids	Young adults, students, cognitively active individuals	Protein isolation + concentration + addition of extracts	Intense, stimulating, citrus-like

**Table 4:** Functional additives in whey-based beverages and their benefits.

Additives	Benefit of Use	Literature Source
Fruit juices (e.g., berry, citrus)	Enhance sensory appeal; increase vitamin C content	[38,129,130]
Chia seeds, matcha, turmeric	Provide omega-3 fatty acids; add anti-inflammatory and metabolic benefits	[87,131]
Probiotic strains (Lactobacillus, Bifidobacterium)	Support digestive health; enhance immune function	[105,124,125,132]
Herbal components	Improve gut health; offer anti-inflammatory effects	[129,133,134]
Medicinal plant extracts	Address vitamin deficiencies; improve bioavailability of nutrients	[81,135,136]
Chamomile, mint, rosehip extracts	Strengthen immune system; provide antioxidant properties	[108,137,138]

## Data Availability

No new data were created or analyzed in this study. Data sharing is not applicable to this article.
